# Discovery of potential anti-infectives against *Staphylococcus aureus* using a *Caenorhabditis elegans* infection model

**DOI:** 10.1186/1472-6882-14-4

**Published:** 2014-01-06

**Authors:** Cin Kong, Wageeh A Yehye, Noorsaadah Abd Rahman, Man-Wah Tan, Sheila Nathan

**Affiliations:** 1 School of Biosciences and Biotechnology, Faculty of Science and Technology, Universiti Kebangsaan Malaysia, 43600 UKM Bangi, Selangor, Malaysia; 2 Nanotechnology & Catalysis Research Centre (NANOCAT), Block 3A, Institute of Postgraduate Studies Building, University of Malaya, Kuala Lumpur, Malaysia; 3 Department of Chemistry, Faculty of Science, University of Malaya, Kuala Lumpur, Malaysia; 4 Department of Genetics and Department of Microbiology and Immunology, Stanford University School of Medicine, Stanford, CA, USA; 5 Current Affiliation: Department of Infectious Diseases Genentech, 1 DNA Way, 11-316 Mail Stop 33, South San Francisco, USA

**Keywords:** *S. aureus*, *C. elegans*, Anti-infectives

## Abstract

**Background:**

The limited antibiotic options for effective control of methicillin-resistant *Staphylococcus aureus* infections has led to calls for new therapeutic approaches to combat this human pathogen. An alternative approach to control MRSA is through the use of anti-infective agents that selectively disrupt virulence-mediated pathways without affecting microbial cell viability or by modulating the host natural immune defenses to combat the pathogen.

**Methods:**

We established a *C. elegans* – *S. aureus* liquid-based assay to screen for potential anti-infectives against *S. aureus*. The assay was utilized to screen 37 natural extracts and 29 synthetic compounds for the ability to extend the lifespan of infected nematodes. Disc diffusion and MIC microdilution tests were used to evaluate the anti-microbial properties of these natural extracts and synthetic compounds whilst *in vivo* bacterial CFU within the *C. elegans* gut were also enumerated.

**Results:**

We screened a total of 37 natural extracts and 29 synthetic compounds for anti-infective properties. The screen successfully revealed 14 natural extracts from six plants (*Nypa fruticans, Swietenia macrophylla, Curcuma longa, Eurycoma longifolia, Orthosiphon stamineus* and *Silybum eburneum*) and one marine sample (*Faunus ater*) that improved the survival of *S. aureus*-infected worms by at least 2.8-fold as well as 14 synthetic compounds that prolonged the survival of *S. aureus*-infected nematodes by 4-fold or greater. An anti-microbial screen of all positive hits demonstrated that 8/28 hits had no effect on *S. aureus* growth. Of these 8 candidates, 5 of them also protected the worms from MRSA infection. We also noted that worms exposed to *N. fruticans* root and *O. stamineus* leaf extracts showed reduced intestinal colonization by live *S. aureus*. This suggests that these extracts could possibly activate host immunity to eliminate the bacteria or interfere with factor/s that prevents pathogen accumulation.

**Conclusion:**

We have successfully demonstrated the utility of this liquid-based screen to identify anti-infective substances that prolong *S. aureus-*infected host survival without affecting bacterial cell viability.

## Background

*Staphylococcus aureus* is capable of causing a variety of human diseases in both hospital and community settings. Diseases caused by *S. aureus* range from superficial skin infections to life threatening systemic bacteremia [1]. The effectiveness of current antibiotic treatments that interfere with *S. aureus* growth and viability is limited by the development of drug resistance. The rapid emergence of antibiotic resistant strains [2,3] highlights the urgent need for newer and safer strategies to combat infection. An alternative approach to overcome the pitfalls of drug resistance is to develop anti-infective agents that selectively disrupt virulence-mediated pathways without affecting microbial cell viability [4-6] or by modulating the host natural immune defenses to combat the pathogen [7].

A number of different strategies have been adopted to identify new anti-infectives and these include using whole-animal infection models such as *Caenorhabditis elegans*[8-12]. Whilst traditional *in vitro* and whole cell drug screens are acknowledged as the established paradigm for identifying antimicrobial molecules, the whole animal approach allows for early and direct assessment of *in vivo* drug efficacy, thus, eliminating compounds that are toxic to the host or with poor pharmacokinetic properties [13]. As current understanding of host-pathogen interactions and bacterial pathogenesis continues to increase [14,15], the *C. elegans* model presents an advantage in being able to simultaneously identify compounds that target bacterial virulence as well as host defense.

In a live animal model with a recognized host-pathogen interaction, potential hits that selectively disrupt the virulence pathways utilized by pathogens to establish infections can be identified [13,16]. Furthermore, several conserved innate immune signaling pathways have been revealed from studies of host-pathogen interactions using *C. elegans*[17]. Conceptually, anti-infectives that target nonessential genes are likely to impose a lower level of selective pressure and probability of resistance development [18] whilst ensuring the preservation of host endogenous microflora.

Infection of *C. elegans* by *S. aureus* is a robust platform to elucidate the mechanisms of host-pathogen interaction [19-22]. In this study, our aim was to extend these studies to the discovery of novel anti-infectives. To achieve this objective, we chose to establish a *C. elegans – S. aureus* liquid-based screen to identify anti-infectives that extend the lifespan of *S. aureus* - infected nematodes from a collection of locally acquired natural products and synthetic compounds.

## Methods

### Bacterial and nematode strains

The methicillin-susceptible *S. aureus* strain NCTC8325-4 [19] and methicillin-resistant *S. aureus* (MRSA) strain ATCC33591 were grown with aeration in Trypticase Soy (TS) media (Oxoid/Pronadisa) at 37°C while *Escherichia coli* strain OP50 was grown in Luria Bertani (LB) broth supplemented with streptomycin (100 μg/mL). The wild type *C. elegans* Bristol N2 hermaphrodite strain was propagated on nematode growth medium (NGM) and fed on the standard laboratory food source, *E. coli* OP50. The animals were age-synchronized by bleaching with alkaline hypochlorite and sodium hydroxide to release embryos. Embryos were placed on plates containing concentrated *E. coli* DH5α at 25°C until the worms reached the young adult stage. To eliminate the confounding effects of reproduction on the scoring of surviving worms, wild type *C. elegans* were made sterile by RNAi knockdown of the *pos-1* gene which resulted in worms laying unhatched eggs [23]. The *pos*-1 RNAi treated worms were grown for ~45 hrs at 25ºC until they reached young adult stage and were ready to be used in the screen.

### Preparation of extracts and compounds

A total of 18 plant species and one marine sample were the source of the natural extracts. Extracts UE-01-1 to UE-08 were extracted according to the previously described method [24]. Briefly, the plant materials and animal sample were collected from several locations in Malaysia. The samples were authenticated, deposited and catalogued with specimen voucher numbers (Additional file 1). All samples were then air-dried, powdered and extracted with polar and non-polar solvents. Solvents were then removed by evaporation using a rotary evaporator. Extracts UE-09 to UE-20 (except UE-15) were prepared by Herbal Science Pte. Ltd., a local pharmaceutical company. In brief, the raw materials were collected and quality assessed through a series of stringent microbial tests as well as heavy metals test using an Atomic Absorption Spectrophotometer (AAS). They were then dried, comminuted or ground into fine powder before water-based extraction was performed. The microbial and trace metal element tests were repeated again on the yield obtained. The finished products were examined for the presence of certain functional groups/molecules using Fourier transform infrared spectroscopy (FTIR) and High-performance liquid chromatography (HPLC). UE-15 was purchased from Merck, Germany.

**Figure 1:**
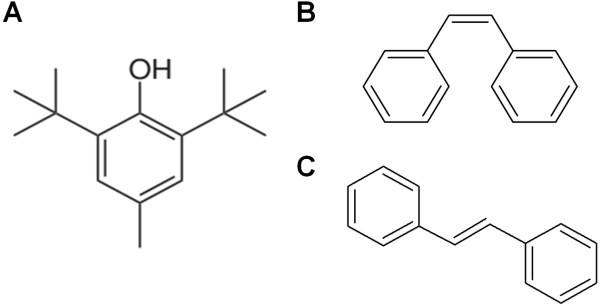
**Chemical structure of butylated hydroxytoluene (BHT) and stilbenes. (A)** BHT, with its IUPAC name 2,6-bis(1,1-dimethylethyl)-4-methylphenol, is chemical derivative of phenol (C_6_H_5_OH) [25]. **(B)** and **(C)** refer to the two isomers of stilbene (IUPAC name: 1,2-diphenylethene). **(B)** (*Z*)-stilbene or *cis*-stilbene and **(C)** (*E*)-stilbene or *trans*-stilbene.

Four different series of butylated hydroxytoluene (BHT) derivatives (Figure 1) were designed and synthesized by the Chemistry Department, University of Malaya, Malaysia. Thiosemicarbazides were synthesized according to the method of Yehye et al. [25,26]. 1,3,4-thiadiazoles and 1,2,4-triazole bearing the free radical scavenger BHT were synthesized using acid-(base-) catalyzed intramolecular dehydrative cyclization reaction of the 1-acylthiosemicarbazides whilst hydrazones were synthesized from the reaction between free amino groups and properative aldehydes (Yehye et al., in preparation). All extracts and compounds were dissolved in dimethyl sulfoxide (DMSO), filtered through a 0.2 μm membrane filter and stored at −20°C. Detailed information on all natural extracts and synthetic compounds used in this study is provided in Additional file 2.

### *C. elegans* survival assay

For the agar-based assay, 10 μL of a *S. aureus* overnight culture in TS broth was spread on 3.5 cm TS agar plates and incubated at 37°C for 24 hrs. The plates were then allowed to equilibrate to room temperature for at least 1 hour before use. A total of 40 young adult worms were transferred to the agar plate and incubated at 25°C. Worm mortality was monitored at 24-hour intervals. *E. coli* OP50 on NGM was used as the negative control. Three independent experiments were performed.

For the liquid-based assay, a 24-well plate was filled with *S. aureus/*MRSA in a liquid medium (80% M9 buffer, 20% *S. aureus* overnight culture and 10 μg/mL cholesterol) and 20 synchronized young adult nematodes were transferred into each well. Wells containing *C. elegans, E. coli* OP50 and M9 buffer served as the control. The total number of worms for each treatment was 120 in six wells representing six technical replicates. The plate was incubated at 25°C and survival was monitored every 24 hrs following exposure to the pathogen.

### Anti-infective screening

Screening was performed in liquid medium in a 24-well plate. The liquid screening medium consisted of worm M9 buffer and overnight culture of *S. aureus*/MRSA in TS broth with 10 μg/mL of cholesterol at a ratio of 4:1 (v/v). The medium was supplemented with either natural extracts or synthetic compounds to a final concentration of 200 μg/mL (natural extracts) and 200 μM (synthetic compounds). All components of the assay were mixed evenly and 500 μL of medium was transferred into each well in a 24-well plate. In control wells, the extract or synthetic compound was replaced with 1% DMSO or *S. aureus* was replaced by *E. coli* OP50 as the nematode food source. Each extract or compound was tested in triplicate wells. Approximately 10–15 age-synchronized *pos-1* treated young adult stage N2 nematodes were transferred into each well and the plate was incubated at 25°C. Worms treated with *pos-1* RNAi produced unhatched eggs thereby eliminating potential complications during the scoring of worms. Worm survival was scored manually every 24 hrs for 5 days. In addition, turbidity of the extract/compound containing media was also observed and recorded. An extract or compound was considered a positive hit if >50% of the infected worms survived compared to ≤ 20% survival in the untreated control worms in least two replicates. All positive hits from the primary screen were subjected to a secondary screen for confirmation. A summary of the one-step anti-infective liquid screen approach used in this study is presented in Additional file 3 and is compared to the previously reported two-step agar-liquid protocol used by Moy et al. [8] for screening of antimicrobials against *Enterococcus faecalis* and high throughput liquid-based chemical screen to screen for molecules that attenuate *Pseudomonas aeruginosa* virulence and rescue *C. elegans* from infection [11].

### Determination of antimicrobial property by disc diffusion assay

The disc diffusion method [27] was used to determine if the extracts and compounds also promoted antimicrobial features. Sterile filter paper discs (Whatman No. 1, 6 mm) were impregnated with each of the extracts (20 mg/mL) or compounds (20 mM) and left to dry under sterile conditions overnight. *S. aureus* was spread evenly onto the surface of TS agar plates using glass beads before the discs were positioned on the inoculated agar surface. The bacterial inoculum size was standardized at 10^6^ cfu/mL by adjusting the optical density of the bacterial suspension. DMSO served as the negative control with gentamicin (200 μg/mL) as the positive control. All plates were incubated for 18 hrs at 37°C. Antibacterial activity was confirmed by the presence of a clear zone of inhibition around the disc.

### Determination of MIC and MBC

The bacteriostatic and bactericidal effects of all positive extracts and compounds were evaluated using the broth microdilution minimum inhibitory concentration (MIC) assay [28]. A range of different concentrations of extract/compound was prepared in a 48-well plate, followed by inoculation with 10^6^ cfu/mL *S. aureus*. The plate was then incubated at 37°C for 18 hrs. Gentamicin, the standard antibiotic for *S. aureus* infections, was used as a control. All extracts/compounds were assayed in triplicate. The MIC endpoint recorded was the lowest concentration of the extract/compound used in wells that showed no turbidity after 18 hrs incubation, indicating an absence of bacterial growth. The presence or absence of turbidity was confirmed by absorption readings at 600 nm. The minimum bactericidal concentration (MBC) was determined by spreading the culture from the respective wells on sterile agar plates. The MBC value was the lowest concentration of extract/compound used where no apparent growth of bacteria was observed on the agar plate.

### Enumeration of bacterial colony forming units (CFU) within the *C. elegans* gut

After 24 hours exposure to *S. aureus* in liquid medium in the presence and absence of extracts/compounds, 10–12 live worms were randomly picked and briefly anesthetized in 25 mM Levamisole. The worms were washed at least twice in 200 μL antibiotic cocktail comprising 25 mM Lev and gentamicin 10 μg/mL followed by incubation for 45 min to 1 hour to completely kill bacterial cells associated with the worm cuticle. Then, the worms were washed three times with 200 μL of 25 mM Lev to eliminate the killed bacteria and residual antibiotic. The number of worms were recorded followed by mechanical disruption in 50 μL of 1% Triton X (Sigma-Aldrich, USA; X100) using a motorized pestle. Serial dilutions were performed on the worm lysates and 10 μL of each dilution was spotted on TS agar supplemented with 5 μg/mL nalidixic acid. Colonies were counted after incubating the plates at 37°C overnight. Bacterial CFU per worm was calculated using the formula reported by Ooi et al. [29].

### Statistical analysis

All data are presented as mean ± standard deviation (SD) of at least two independent experiments. All screening assays, antibacterial tests and *in vivo* CFU assay were replicated in a comparable manner. Data from the killing assays were analyzed with StatView® 5.0.1 (SAS Institute, Inc) and plotted using the Kaplan-Meier Cumulative Survival Plot for Time (nonparametric survival analysis). The pair wise comparison was analyzed using the Log-rank (Mantel-Cox) significance test.

## Results

### Liquid-based screen was able to detect more hits

**Figure 2:**
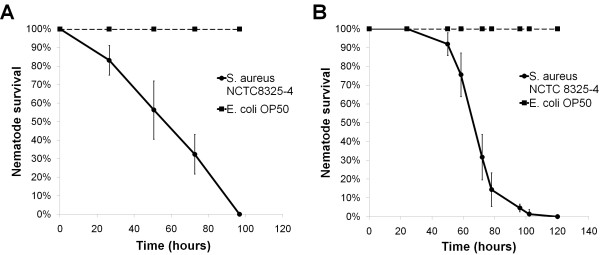
***S. aureus *kills *C. elegans *in both liquid and agar medium.** Representative survival curves of *C. elegans* following infection by *S. aureus* strain NCTC8325-4 in **(A)** liquid medium consisting of 80% M9 buffer, 20% *S. aureus* overnight culture and cholesterol and **(B)** TS agar medium. Graph **(A)** shows the mean ± SD of six replicates (20 nematodes/replicate) and graph **(B)** presents the mean ± SD of three replicates (40 nematodes/replicate) from a representative of three independent assays.

Most studies on *C. elegans* infections by *S. aureus* are routinely performed on agar plates. In this study, we observed significant reduction in the lifespan of *C. elegans* infected by *S. aureus* in both the agar-based and liquid-based assays (Figure 2A and B). Specifically, the mean time to death (TD_mean_) for *S. aureus*-infected animals assayed on agar was 73 ± 1.5 hrs which was consistent with previous studies [20]. Similarly, in the liquid-based assay, worms in M9 buffer and *S. aureus* culture (4:1, v/v) had a TD_mean_ of 72 ± 5 hrs. *S. aureus* required 4 to 5 days for complete killing of *C. elegans*. In liquid medium, the worms infected by *S. aureus* started to die after 24 hrs exposure to the pathogen. Mobility, pharyngeal pumping and foraging rates of worms exposed to pathogens progressively decreased with time until all worms became immobile and died. The results confirmed that in liquid medium, *S. aureus* is able to kill the nematodes as efficiently as the standard agar-based assay system.

**Figure 3:**
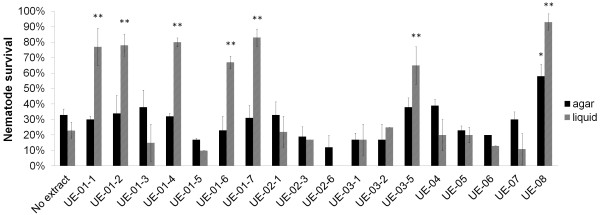
**Liquid-based assay identified more hits than the agar-based assay.** Comparison of hits from the preliminary screen of 18 natural extracts on both agar-based and liquid-based assays. All natural extracts were tested at same concentration (200 μg/mL) in both agar and liquid assays. The graph represents the mean percentage of survival ± SD at 72 hours post-infection of three replicates from a representative of two individual experiments. The survival of extract-treated worms is compared to the control (no extract). (*) indicates positive in agar-based screens while (**) indicates positive in liquid-based screens.

To establish the utility of the liquid assay for anti-infective screening, we screened a subset of 18 natural extracts using both liquid- and agar-based assays. In the agar-based assay, extracts were added into the TS agar whilst the prepared agar was in a molten state. Nematode survival on extract-added plates was compared to the control (agar without extract). Only 1/18 demonstrated potential anti-infective properties when compared to controls in the absence of extract on solid agar (Figure 3). By contrast, when the assay was performed in the liquid-based assay, we were able to detect 7/18 potential hits that reproducibly extended the lifespan of *S. aureus*-infected worms in at least two independent experiments (Figure 3). These results indicated that screening using a liquid-based assay was able to detect more hits than the conventional agar-based assay. Thus, we carried out the anti-infective screen on the rest of the extracts and compounds using the liquid-based screen.

Direct comparison of the hits identified from both the liquid and agar based screens (Figure 3) confirmed that only UE-08 promoted the survival of infected worms in both agar-based and liquid based screens. UE-01-1, UE-01-2, UE-01-4, UE-01-6, UE-01-7 and UE-03-5 were able to improve the lifespan of infected worms in liquid medium but not in agar and this could be due to thermal degradation of certain active molecules (saponins, flavonoids) during the media preparation. Further comparisons between the solid agar and liquid-based screens are elaborated in Table 1.

**Table 1:** Comparison between agar-based versus liquid-based screens

**Criteria**	**Agar-based**	**Liquid-based**
Positive hits identified	1 out of 18 natural extracts screened	7 out of 18 natural extracts screened
Average time for media preparation	2-3 days	<30 minutes
Possibility of compound degradation	Addition of compound into the molten agar at high temperature may cause compound degradation	Does not involve high temperature. Thus, degradation of compound is not possible
Exposure to compound	Compound is restricted to the agar media, resulting in limited worm exposure to the compound	Worms are bathed in a homogenous solution of compound
Counting efficiency	Time-consuming to locate the worms on a larger surface area	Smaller surface area and clear distinguishable phenotype of the worms allows quick scoring
	Possible agar color changes complicate worm scoring	Colorless M9 buffer eases the scoring of worms

The advantages of the liquid-based screen developed are listed along with the limitations of an agar-based assay.

### Selected extracts and compounds enhanced the survival of *S. aureus*-infected nematodes

**Figure 4:**
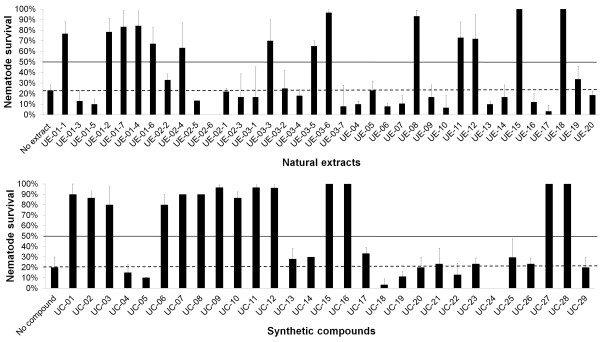
**Extracts and compounds rescued the worms from a *S. aureus *infection.** Percentage of survival for *S. aureus*-infected nematodes upon exposure to extracts (200 μg/mL) and compounds (200 μM) in the primary anti-infective screen. The graph depicts the percentage of survival at 72 hours post-infection when the assay was carried out in the absence of presence of different extracts/compound. Results are expressed as mean ± SD from a representative of at least two individual assays. Dashed lines show the percentage of survival for untreated worms (no extract/no compound) while straight lines demarcate the positive hits. Extracts and compounds that contributed to > 50% infected worm survival were considered as positive in the primary screen.

We performed a small scale screen in 24-well plates using the *S. aureus* – *C. elegans* liquid infection assay to identify more potential anti-infectives that improved the survival of infected worms. Screening over 37 natural plant and marine invertebrate extracts and 29 synthetic compounds revealed a total of 14 natural extracts and 14 synthetic compounds that conferred increased survival of *S. aureus*-infected nematodes (Figure 4). The criteria adopted to classify an extract or compound as a positive hit was the survival of >50% of the infected worms compared to ≤ 20% survival in untreated wells over at least two replicates. All positive hits contributed to more than 60% *C. elegans* survival following infection and treatment for 72 hrs compared to about 20% survival in untreated controls (Table 2). All positive natural extracts increased nematode survival by 2.8-fold or more while all positive synthetic compounds exhibited a more significant increase in survival of at least 4-fold. The worms fed on *E. coli* OP50 remained alive throughout the assay. Detailed information on all positive hits is shown in Table 2.

**Table 2:** **Natural extracts and synthetic compounds that promote survival of *S. aureus*-infected nematodes**

**No.**	**Extract/compound ID**	**Plant/animal species**	**Extraction solvent**	**Mean % of survival**	**Fold change survival vs. untreated**^**a**^
1	UE-01-1	*Nypa fruticans* husks	Methanol	76.7	3.3
2	UE-01-2	*N. fruticans* leaves	Hexane	78.4	3.4
3	UE-01-4	*N. fruticans* roots	Butanol	84.2	3.7
4	UE-01-6	*N. fruticans* roots	Chloroform	67.3	2.9
5	UE-01-7	*N. fruticans* leaves	Chloroform	83.3	3.6
6	UE-02-4	*Faunus ater*	Methanol	63.3	2.8
7	UE-03-3	*Swietenia macrophylla* seeds	Methanol	70.0	3
8	UE-03-5	*S. macrophylla* seeds	Ethyl acetate	65.0	2.8
9	UE-03-6	*S. macrophylla* seeds	Butanol	96.7	4.2
10	UE-08	*Curcuma longa*	Water	93.3	4
11	UE-11	*Eurycoma longifolia* roots	Water	73.0	3.2
12	UE-12	*Orthosiphon stamineus* leaves	Water	71.8	3.1
13	UE-15	Curcumin	-	100.0	4.3
14	UE-18	*Silybum eburneum* seeds	Water	100.0	4.3
15	UC-01	-	-	90.0	4.5
16	UC-02	-	-	86.7	4.3
17	UC-03	-	-	80.0	4
18	UC-06	-	-	80.0	4
19	UC-07	-	-	90.0	4.5
20	UC-08	-	-	90.0	4.5
21	UC-09	-	-	96.7	4.8
22	UC-10	-	-	86.7	4.3
23	UC-11	-	-	96.7	4.8
24	UC-12	-	-	96.3	4.8
25	UC-15	-	-	100.0	5
26	UC-16	-	-	100.0	5
27	UC-27	-	-	100.0	5
28	UC-28	-	-	100.0	5

^a^Note that ~20% of the untreated worms survived 72 hours post-infection. The mean percentage of survival from one representative biological replicate out of three independent assays is presented.

**Figure 5:**
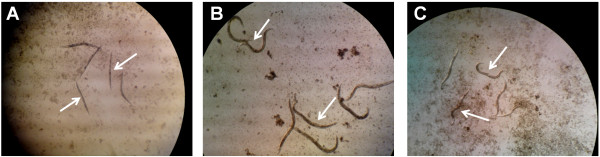
**Distinct appearances of dead and alive *C. elegans*.** Representative micrographs of *C. elegans* exposed either to *S. aureus ***(A)**, *E. coli* OP50 **(B)** or *S. aureus* in the presence of extracts/compounds **(C)**. The worms were examined under a stereomicroscope (Nikon SMZ645) at a magnification of x40. White arrows denote the worms.

The striking differences in the appearance of live worms and worms that succumbed to infection facilitated the screening process. The infected worms were generally smaller in size as compared to healthy worms fed on *E. coli* OP50. Dead worms did not move or exhibit muscle tone and did not respond to external stimuli. They appeared as straight rigid rods in the medium with no observable pharyngeal pumping (Figure 5A). In contrast, worms exposed to *E. coli* OP50 maintained a sinusoidal ‘S’ shape and moved actively. These worms grew in size and developed into gravid adults which produced eggs (Figure 5B). With the addition of extract/compound that exhibited potential anti-infective properties, the worms were rescued from the *S. aureus* infection as demonstrated by their ability to overcome the debilitating effects of the infection. As indicated in Figure 5C, the surviving infected nematodes appear to be smaller in size, similar to that of the untreated infected worms. Nevertheless, the live worms were able to maintain the sinusoidal ‘S’ shape posture and respond to external stimuli when touched. In addition, the worms continued to move actively even after 72 hrs exposure to *S. aureus*. Although the worms were able to survive upon *S. aureus* infection in the presence of hit extracts/compounds, most of the live worms appeared to experience delayed development as compared to healthy worms fed on *E. coli* OP50.

Among 18 plant species tested, extracts from six plants (*N. fruticans, S. macrophylla, C. longa, E. longifolia, O. stamineus* and *S. eburneum*) were able to extend the lifespan of *S. aureus*-infected animals. The marine sample (*F. ater*) also possessed potential anti-infective activity. *N. fruticans, S. macrophylla, C. longa, E. longifolia* and *O. stamineus* are commonly found in Malaysia and used widely by the local community as folk medicine. Hence, local traditional herbs are a promising source of largely unexplored classes of anti-infectives.

We tested seven samples from *N. fruticans* obtained from different parts of the plant through the use of different extraction solvents. Our screen identified 5/7 *N. fruticans* samples that increased the survival of infected nematodes by 2.9 to 3.7 fold. Different parts of the plant, for example, leaves, roots and husks, may contain different sets of bioactive compounds whilst the different extraction solvents used may also separate different bioactive constituents. Alternatively, the differences may be due to variations in the quantity of the individual active components in these tissues. Both *C. longa* and curcumin showed an equally strong effect by enhancing the survival of *S. aureus*-infected nematodes by at least 4-fold (Table 2). Curcumin is the main active ingredient in *C. longa* suggesting that curcumin might be the contributing bioactive component of *C. longa*. Methanol, butanol and ethyl acetate extracts of *S. macrophylla* were also able to prolong the lifespan of *S. aureus*-infected worms with the butanol extract showing the most enhanced effect, whereby worm survival increased by 4.2-fold. Of all the extracts tested, the *S. macrophylla* butanol extract, curcumin and *S. eburneum* seed extract demonstrated very promising anti-infective activity by promoting the survival of *S. aureus*-infected worms by more than 4-fold.

**Figure 6:**
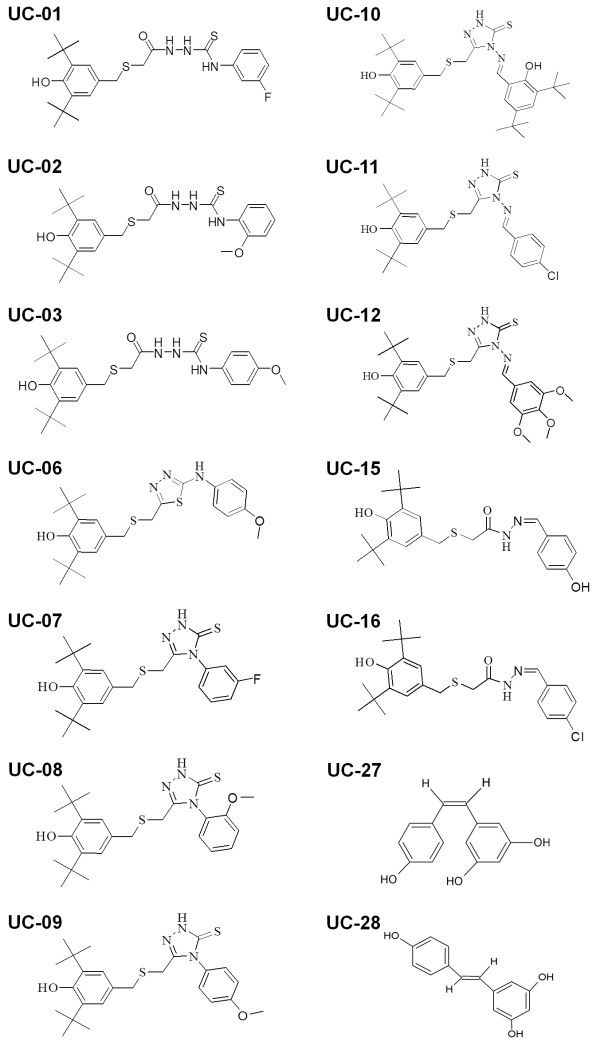
**Structure of the compounds that promoted the survival of *S. aureus *infected nematodes.** Compounds UC-01, UC-02 and UC-03 are thiosemicarbazides, UC-06 is 1,3,4-thiadiazoles, UC-07, UC-08 and UC-09 are 1,2,4-triazole-5-thiones, UC-10, UC-11 and UC-12 are 4-(substituted benzylideneamino-1,2,4-triazole-5-thiones, UC-15 and UC-16 are hydrazones and UC-27 and UC-28 are stilbene derivatives. All compounds, with the exception of the stilbene derivatives, are fused to a BHT moiety.

With the synthetic compounds, all 14 hits promoted the survival of infected worms by at least 4-fold with UC-15, UC-16, UC-27 and UC-28 demonstrating the most prominent effect, whereby worm survival increased by 5-fold. Compounds UC-01, UC-02 and UC-03 are variations of thiosemicarbazide, UC-07, UC-08, UC-09, UC-10, UC-11 and UC-12 were synthesized based on of 1,2,4-triazole-5-thiones, UC-15 and UC-16 are derived from hydrazones, UC-27 and UC-28 are stilbene derivatives while UC-06 is a variant of 1,3,4-thiadiazole. With the exception of stilbene, all other compounds were fused to a BHT moiety. The structures of all 14 positive synthetic compounds are shown in Figure 6.

**Figure 7:**
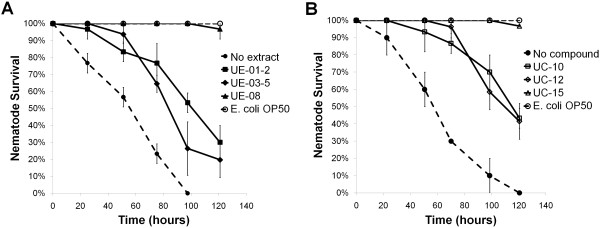
**Survival of *C. elegans *is improved over time upon supplementation of extracts and compounds. (A and B)** The survival curves of *S. aureus*-infected *C. elegans* upon exposure to selected hit extracts (200 μg/mL) and compounds (200 μM). Enhanced survival of infected worms can be seen in the presence of extracts UE-01-2, UE-03-5, UE-08 **(A)** and compounds UC-10, UC-12 and UC-15 **(B)**. Data at each time point are the average of three wells with each well containing approximately 10–15 animals per well. In a pair-wise comparison to no extract using log-rank tests, the difference between all of the treatments was significant (*p* < 0.0001).

As illustrated in Figure 7, different extracts/compounds exhibited different degrees of protection in extending the lifespan of nematodes following *S. aureus* infection. During the screening process, we also took note of changes in media turbidity indicative of bacterial presence and growth. Upon addition of selected extracts and compounds, for example UE-08 and compound UC-15, into the medium, a significant clearing of the medium was noted. When we correlated this observation to the MIC and MBC results of all positive hits as shown in Table 3, we noted that extract UE-08 and compound UC-15 possessed strong antibacterial activity against *S. aureus*. The survival curves presented in Figure 7A and B reflect that both these hits exerted a strong protective effect on the worms and ~100% of worms were rescued from the lethal infection throughout the experiment (over 5 days). Based on this, we propose that UE-08 and UC-15 extend the lifespan of worms during a *S. aureus* infection through their antimicrobial activity against *S. aureus*.

**Table 3:** ***In vitro *** **MIC and MBC values of all positive extracts (A) and compounds (B) against *S. aureus***

**(A) No.**	**Extract ID**	**Standard medium (TS broth)**		**Screening medium (20% TS broth + 80% M9)**	
		**Concentration (μg/mL)**		**Concentration (μg/mL)**	
		**MIC**	**MBC**	**MIC**	**MBC**
1	UE-01-1	<125	1000	<125	500
2	UE-01-2	2000	-	2000	-
3	UE-01-4	-	-	-	-
4	UE-01-6	2000	-	2000	-
5	UE-01-7	1000	2000	500	2000
6	UE-02-4	-	-	-	-
7	UE-03-3	-	-	-	-
8	UE-03-5	-	-	-	-
9	UE-03-6	-	-	-	-
10	UE-08	<125	<125	<125	<125
11	UE-11	-	-	-	-
12	UE-12	-	-	-	-
13	UE-15	1000	2000	500	2000
14	UE-18	1000	-	500	-
**(B) No.**	**Compound ID**	**Standard medium (TS broth)**		**Screening medium (20% TS broth + 80% M9)**	
		**Concentration (μM)**		**Concentration (μM)**	
		**MIC**	**MBC**	**MIC**	**MBC**
1	UC-01	125	500	125	500
2	UC-02	2000	2000	2000	2000
3	UC-03	2000	2000	1000	2000
4	UC-06	2000	2000	1000	2000
5	UC-07	200	1000	125	1000
6	UC-08	1000	2000	1000	2000
7	UC-09	2000	2000	1000	2000
8	UC-10	-	-	-	-
9	UC-11	200	-	200	-
10	UC-12	1000	-	1000	-
11	UC-15	<62.5	<62.5	<62.5	<62.5
12	UC-16	2000	-	1000	-
13	UC-27	200	200	200	200
14	UC-28	200	200	200	200

The negative (DMSO) and positive controls (gentamicin) were run in parallel in all replicates. The MIC of gentamicin towards *S. aureus* was 1.2 μg/mL and MBC was 2.4 μg/mL.

MIC, minimum inhibitory concentration and MBC, minimum bactericidal concentration.

In contrast, upon supplementation of the other selected extracts and compounds into the screening media, we observed a cloudy appearance (similar to the untreated control) of the media indicating continued bacterial growth. We confirmed our observations by measuring the turbidity at 600 nm. Nevertheless, addition of these extracts and compounds significantly delayed the killing of worms by *S. aureus*. Data for lifespan extension by representative hits (extracts UE-01-2 & UE-03-5 and compounds UC-10 & UC-12) that exhibited this phenomenon are presented in Figure 7. All these hits either did not kill *S. aureus* or only inhibited *S. aureus* growth at very high concentrations (Table 3). As they did not affect *S. aureus* viability at the tested concentrations, we propose that extracts UE-01-2 & UE-03-5 and compounds UC-10 & UC-12 enhance the survival of *S. aureus*-infected worms using other distinct mechanisms such as reducing bacterial virulence and/or by enhancing host immunity.

### Positive hits exhibited different anti-*S. aureus* activity

**Figure 8:**
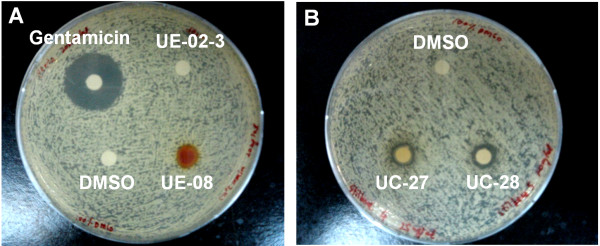
**Inhibition of *S. aureus *growth by extracts and compounds.** The clear zone formed around the discs indicated that *S. aureus* was susceptible to extract UE-08 **(A)** and compounds UC-27 & UC-28 **(B)**. The negative control (DMSO) showed no clear zone around the disc while the positive control (200 μg/mL gentamicin) suppressed the growth of *S. aureus* and formed a 20 mm zone of inhibition around the disc.

The disc diffusion assay and MIC test were used to evaluate the positive hits obtained from the anti-infective screen for their ability to inhibit *S. aureus* growth. The disc diffusion assay detected two natural extracts (UE-08 & UE-15) and four synthetic compounds (UC-01, UC-15, UC-27 and UC-28) out of 28 positive hits, with antibacterial effect on *S. aureus* (Figure 8). To further validate the hits, we performed the broth microdilution MIC test to obtain both MIC and MBC values.

The respective MIC and MBC values of all 28 positive hits in standard medium (TS broth) and screening medium (20% TS broth + 80% M9 medium) are presented in Table 3A and B. The negative control (DMSO) and positive control (gentamicin) were run in parallel with a recorded MIC for gentamicin towards *S. aureus* at 1.2 μg/mL and MBC of 2.4 μg/mL. As the clearing of the liquid medium has been used as a criterion during the anti-infective screening, we also performed the MIC microdilution test using the screening medium. We found relatively similar values for both media tested. Of the positive hits screened, two natural extracts (UE-01-1 and UE-08) were found to inhibit *S. aureus* growth at a concentration of < 200 μg/mL while six compounds (UC-01, UC-07, UC-11, UC-15, UC-27 and UC-28) exhibited bacteriostatic effects at a concentration of ≤ 200 μM. Of note is compound UC-15, the most potent antimicrobial agent which suppressed *S. aureus* growth at concentrations less than 62.5 μM in both standard medium and screening medium. In the anti-infective screens discussed above, both 200 μg/mL natural extracts and 200 μM synthetic compounds were the working concentrations used. A further five natural extracts (UE-01-2, UE-01-6, UE-01-7, UE-15 and UE-18) and seven synthetic compounds (UC-02, UC-03, UC-06, UC-08, UC-09, UC-12 and UC-16) affected *S. aureus* growth *in vitro* but at ~ 2-fold higher concentrations than that used in the screen. Interestingly, seven natural extracts (UE-01-4, UE-02-4, UE-03-3, UE-03-5, UE-03-6, UE-11 and UE-12) and one synthetic compound (UC-10) which enhanced the survival of *S. aureus*-infected nematodes, did not exhibit any bacteriostatic or bactericidal activity on *S. aureus* growth *in vitro*. Collectively, the *C. elegans* model-based screen identified eight hits (29%) that inhibited *S. aureus* growth at concentrations lower than that used in the screens, 12 (42%) hits that suppressed *S. aureus* growth at concentrations at least two-fold higher than the screen concentrations and eight hits (29%) that had no effect on *S. aureus* growth *in vitro* even though they were effective in curing the worms from infection.

Both *C. longa* and curcumin demonstrated anti-*S. aureus* properties at different concentrations with *C. longa* inhibiting *S. aureus* growth at a lower concentration (<125 μg/mL) than curcumin (500 μg/mL). This suggests that the *C. longa* extract may contain multiple bioactive compounds that target *S. aureus*. Out of five *N. fruticans* positive samples, 4 of 5 were capable of inhibiting *S. aureus* growth with the husk methanol extract exhibiting the strongest activity.

All synthetic compounds, with the exception of UC-10, demonstrated antibacterial activity towards *S. aureus* at different concentrations. A comparison between all 14 hits and antibiotics currently used in a clinical setting did not reveal any structural relationship. However, some of the variants from these groups of compounds have previously been reported as potential antibacterials. UC-01, UC-02 and UC-03 are thiosemicarbazides with different elements attached to different positions of the second benzene ring and thiosemicarbazide derivatives have known antimicrobial and antifungal properties [30,31]. Derivatives of thiadiazoles and triazole-thiones (UC-06, UC-07, UC-08, UC-09, UC-11 and UC-12) also showed an inhibitory effect towards *S. aureus* at concentrations ranging from 200 – 2000 μM. Compounds containing a 1,3,4-thiadiazole nucleus and 1,2,4-trizole-thiones have a wide range of pharmacological activities including potential antimicrobial properties [32,33]. On the other hand, hydrazone derivatives are also active against *S. aureus* and *P. aeruginosa*[34]. Natural stilbenes are typical plant metabolites (e.g. resveratrol in grapes) which play a role in defending the host against microbial attack [35]. Stilbene variants such as hydroxystilbene and isopropylstilbene are potential antimicrobials and show a synergistic effect when applied together with antibiotics to inhibit and kill bacteria [36]. Our data consistently demonstrated that both stilbene derivatives (UC-27 and UC-28) showed bactericidal effect towards *S. aureus* at 200 μM. Despite ample evidence indicating the antimicrobial activity of the derivatives of these compounds, the exact mechanism on how these molecules inhibit/kill bacteria remains unknown.

### Positive hits also rescued worms from MRSA infection

From the anti-infective screen and antimicrobial susceptibility test, we identified seven natural extracts (UE-01-4, UE-02-4, UE-03-3, UE-03-5, UE-03-6, UE-11 and UE-12) and one synthetic compound (UC-10) which significantly increased survival of infected nematodes without affecting *S. aureus* replication. The *S. aureus* strain NCTC8325-4 used in the screen is a methicillin-sensitive *S. aureus* (MSSA) [37]. To further explore the potency of these eight candidates, we extended our study by determining their ability in promoting the survival of MRSA-infected worms.

**Figure 9:**
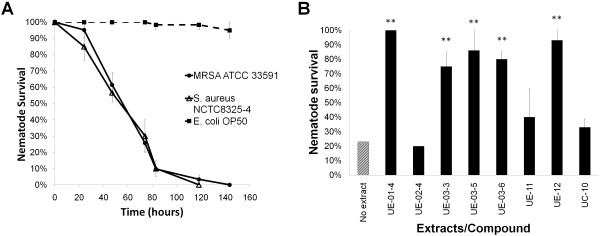
**Nematodes infected by MRSA are rescued by natural extracts. (A)** Kinetics of the killing of *C. elegans* by MRSA ATCC33591, *S. aureus* NCTC-8325-4 and *E. coli* OP50 in liquid medium. Graph shows the mean ± SD of six replicates (20 worms / replicate) from a representative of three independent experiments. **(B)** Survival rate of MRSA-infected nematodes upon exposure to selected extracts (200 μg/mL)/compound (200 μM). Graph shows the percentage of survival at 72 hours post-infection when the assay was carried out without any treatment or treatment with different extracts/compound. Results are expressed as mean ± SD from a representative of two individual assays. (**) denotes significant difference in percentage of survival between untreated (no extract) and treated animals (*p* < 0.005).

As the susceptibility of *C. elegans* towards MRSA strain ATCC33591 has not been studied before, we performed the survival assay in liquid medium to assess the virulence of this MRSA strain towards wild-type *C. elegans*. Our results showed that MRSA ATCC33591 killed all nematodes after 5 days post-infection with a TD_mean_ of 68 ± 3.2 hrs which is comparable to the MSSA strain NCTC8325-4 (Figure 9A). Interestingly, when we evaluated the eight hits from the MSSA screen, 5/8 were also able to rescue the worms from MRSA infection. The five natural extracts that significantly promoted the survival of MRSA-infected nematodes after 72 hours infection were UE-01-4, UE-03-3, UE-03-5, UE-03-6 and UE-12 (*p <* 0.005). All five extracts contributed to more than 70% *C. elegans* survival upon MRSA infection compared to almost 80% death of untreated worms (Figure 9B). Specifically, the five extracts that protected the worms from both antibiotic-sensitive and antibiotic-resistant *S. aureus* infection were extracted from *N. fruticans* roots, *S. macrophylla* seeds and *O. stamineus* leaves.

The survival of MRSA-infected nematodes exposed to UE-02-4, UE-11 and UC-10 was less than 40% (Figure 9B) indicating that these three hits were only efficacious for MSSA but not MRSA infections. Based on this, we suggest that these compounds may be able to render the antibiotic-sensitive strain less virulent but not the antibiotic-resistant strain. It was possible that these hits function as a specific inhibitor of virulence determinants that are complex and strain specific.

### Two natural extracts caused reduction in intestinal bacterial loads

The killing of *C. elegans* by *S. aureus* is correlated with the ability of the bacteria to colonize the worm intestine [20]. *S. aureus* do not persist in the host, yet when the host has been in contact with the bacteria for an adequate period of time, death is observed. Similarly, *Pseudomonas aeruginosa* PA14 also colonize and distend the worm intestinal lumen but do not persist within the host [38]. To address the question of whether the level of live *S. aureus* in the worm intestine remained the same in the presence of extracts, we measured the number of bacteria by performing a CFU assay. *In vivo* CFU counts were enumerated at 12 and 24 hours when >70% of the infected non-treated population remained alive. For this assay, we tested the eight hits that showed no interference with *S. aureus* viability in the antimicrobial test.

**Figure 10:**
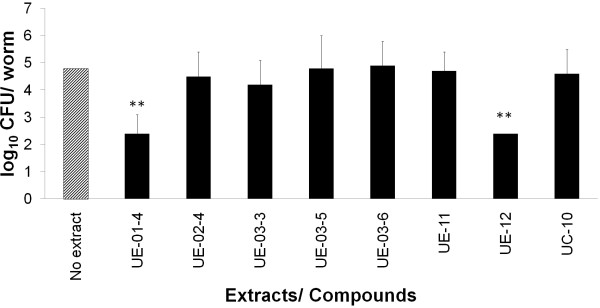
**Extracts suppressed the number of *in vivo S. aureus *in infected animals.** Bacterial CFU in the intestine of *S. aureus*-infected worms were enumerated at 24 hours post-infection in the presence and absence of extracts/compound. All extracts were tested at 200 μg/mL and compound was tested at 200 μM. The bars correspond to mean ± SD of the number of CFU per worm from one representative of two independent replicates. (**) marks the significant difference of *in vivo* CFU counts between untreated (no extract) and treated worms (*p* < 0.0001).

In the absence of extract, *S. aureus* grossly colonized the worm intestine and the *in vivo* CFU counts for *S. aureus*-infected worms was 6.25 × 10^4^ bacteria per worm after 24 hours exposure to the pathogen. This result is consistent with the data reported for worms exposed to PA14, where the bacterial CFU counts can reach up to 10^3^-10^5^ bacteria per worm [29]. Upon supplementation with extract UE-01-4 and UE-12, we observed a significant reduction in the number of bacteria harvested from the *C. elegans* intestine, 2.5 × 10^2^ and 2.3 × 10^2^ CFU/worm respectively (*p <* 0.0001) (Figure 10). One possible explanation for this is that the ingestion of bacteria is abated as a consequence of reduced feeding following exposure to a specific bioactive molecule. We tested this hypothesis by counting the pharyngeal pumping rate of the worms in the presence of UE-01-4, as an indirect measure of the intake of bacteria. In the presence of UE-01-4, pharyngeal pumping rates were similar to untreated worms (Additional file 4). Hence, suffice to say, the low number of intestinal bacteria is not a consequence of a decrease in bacteria consumption, but rather, due to other *in vivo* factor/s that suppress the number of bacteria in the host intestine. For the other five extracts (UE-02-4, UE-03-3, UE-03-5, UE-03-6, UE-11) and compound UC-10, we did not observe any significant difference in the intestinal bacterial loads compared to the untreated animals.

The extracts from *N. fruticans* root (UE-01-4) and *O. stamineus* leaves (UE-12) limited the number of *S. aureus* in the host intestine, suggesting that the mode of action of these extracts likely involved activation of host immunity to reduce the bacterial numbers and thus clear the infection. For extracts UE-02-4, UE-03-3, UE-03-5, UE-03-6, UE-11 and compound UC-10, the manifestation of a healthy worm population that remained colonized by *S. aureus* proposed that these extracts/compound might have rendered the bacteria less virulent and therefore harmless. The less virulent bacteria continued to colonize the host intestine but were unable to bring about death of the host. Alternatively, they may have also modulated the host response to tolerate chronic innocuous intestinal colonization. Both protective outcomes (healthy worms with harmless infection or healthy worms with cleared infection) may arise from either impairing the bacterial virulence or upregulating the host defenses.

## Discussion

We have successfully established a liquid-based *C. elegans* - *S. aureus* anti-infective screen platform which is relatively easy to perform, time-efficient and easily adaptable for large-scale screening of compounds of interest. This simple live animal screen enabled us to identify 14 natural extracts (from eight different plants and one marine sample) and 14 synthetic compounds that significantly extended the survival of *S. aureus*-infected worms. We also observed that five of the selected natural extracts conferred a survival advantage for nematodes infected by MRSA. In agreement with previously reported findings [8,9], our results demonstrate that this screen identifies not only substances with anti-bacterial properties, but also hits that do not affect bacterial replication *in vitro* which are usually overlooked in traditional *in vitro* cell-culture based screens.

We noticed the *S. aureus*- infected worms under both treated and untreated conditions appear smaller in size, similar to previously reported observations on solid-medium [22]. It is less likely that the small size of infected-worms is a result of starvation as shown by previous transcriptome analysis on fasting-response genes [22]. The smaller size of the infected worms may be a consequence of energy conservation and delayed development to compensate for the effort in fighting off the infection. Unlike the uninfected population fed on *E. coli* OP50, we observed that the surviving infected worms in the presence of most hits were also small in size and slow in development. As indicated in the antimicrobial tests, most of the positive hits did not interfere with the growth of bacteria at the concentration used in anti-infective screening (200 μg/mL). Due to the continuous exposure to pathogen in the screening medium, it is not surprising that the live worms resembled the phenotype of infected worms, instead of the phenotype of healthy worms fed on their natural food source.

In contrast to *Salmonella typhimurium* that persistently colonizes and proliferates in the nematode gut [39], *S. aureus* causes transient infection and continuous exposure up to a certain period of time is necessary to achieve maximal worm killing. *S. aureus* can grossly colonize and distend the worm intestinal lumen but does not persist within the infected host [20]. Therefore, we modified the method of Moy et al. [8] by exposing worms to live bacteria throughout the assay. A similar approach where the worms were not pre-infected has been used to screen a small molecule library for compounds that alleviate *P. aeruginosa*-mediated killing [11]. In addition, we were able to simultaneously evaluate compounds or extracts that inhibit bacterial growth and promote host survival by observing the turbidity of the liquid medium. This in turn, enables a quick selection of hits that do not affect bacterial proliferation yet enhance host survival, which will likely yield alternative therapeutics that circumvent bacterial resistance and potentially overcome the limitations of traditional antibiotics.

Among the 28 molecular entities that protected *C. elegans* from lethal infection by *S. aureus*, seven natural extracts and 13 synthetic compounds contain antibacterial properties against *S. aureus* at different concentrations. As expected, both *C. longa* and curcumin were able to inhibit *S. aureus* growth as previously described [40,41]. On the other hand, *S. macrophylla* leaves were previously reported to be active against *S. aureus*[42] but we show that the seed extracts did not affect *S. aureus* growth. Notably, some extracts and compounds demonstrated antibacterial effect at very high concentration (MIC: 2000 μg/mL). Although they are potentially interesting as ‘probe’ molecules, these compounds have limited potential for commercial drug development in their current form.

Of all synthetic compounds tested, 24/29 contain a BHT moiety (Figure 1) in their chemical structure [25]. Since the compounds tested are related to this structure, it is not surprising that the hit rate was high among the synthetic compounds. However, these hits do exhibit different activities suggesting that they may have multiple targets, some of which are not in common and to which they exhibit differential affinity. Up to 13/14 of the hits demonstrated either bacteriostatic or bactericidal effects against *S. aureus* at different concentrations. BHT is a phenolic antioxidant which consists of a hydroxyl group (−OH) bonded directly to an aromatic hydrocarbon group. Other substances that exert comparable free radical scavenging anti-oxidant activity as BHT have been reported to have good antibacterial and antifungal activity [43,44]. Phenolic compounds have also been reported to exhibit antibacterial activity towards gram positive and gram negative bacteria [45,46]. The mechanism thought to be responsible for phenolic toxicity to bacteria includes forming hydrogen bonds with vital proteins such as microbial enzymes [47]. Thus, the anti- *S. aureus* property of all the synthetic compounds tested in this study may be associated with the presence of the phenolic moiety in the BHT chemical structure. Moreover, the backbone structures of the compounds (thiosemicarbazides, thiadiazoles, triazole-thiones and hydrazones) have been reported to possess antimicrobial activity against both gram positive and negative bacteria [31,32,34]. This is believed to be able to elevate the antimicrobial properties of these compounds.

Among all synthetic compound hits, UC-10 appeared to be particularly interesting as it rescued the infected worms without affecting *S. aureus* growth. UC-10 is a derivative of 4-(substituted benzylideneamino)-1,2,4-triazole-5-thione. In terms of structure, UC-10 differs from UC-11 and UC-12 at the second benzene ring, at which a –OH group was substituted at the ortho position. The different activity of this compound, as compared to the rest of the compounds, may be attributed to the presence of this additional –OH group and two butyl groups, resulting in two –OH groups flanking by tert-butyl groups into one structure (Figure 6). Modification of this aromatic ring diminished the antibacterial property of the 4-(substituted benzylideneamino)-1,2,4-triazole-5-thiones compound fused to the BHT moiety. Unexpectedly, this has resulted in its ability to rescue the infected host via a distinct mechanism, probably by targeting bacterial virulence or host immunity.

Some extracts and compounds were able to promote nematode survival at concentrations much lower than their respective MIC values. The identification of seven extracts and one compound that do not interfere with bacteria replication suggests that this group of substances may apply non-conventional strategies to permit host survival whilst not affecting cell viability. These compounds may attenuate bacterial factors essential for infection such as virulence determinants involved in host damage and disease [4]. Additionally, some of these substances could modulate host responses towards the pathogen [48] or act as an immunostimulator [49] that induces the host immune defense to get rid of infection.

One of the most significant findings is the three different extracts from *S. macrophylla* seed (UE-03-3, 03–5, 03–6) that prolonged the survival of both *S. aureus* and MRSA-infected nematodes without diminishing the degree of *S. aureus* accumulation in the *C. elegans* gut. A similar effect was demonstrated in the study by Dharmalingam et al. [24] where the ethyl acetate extract (UE-03-5) also protected *C. elegans* from a lethal *P. aeruginosa* infection without affecting bacterial viability and *in vivo* proliferation of PA14 [24]. The fact that this extract permitted the surviving host to harbor a substantial bacterial load in the gut following infection by *S. aureus* and *P. aeruginosa* indicates that this extract might have attenuated a bacterial virulence factor or pathway which is common among pathogens. Hence, the anti-infectives identified using the *C. elegans* screen may be multifunctional and able to target a broad range of human pathogens. With the existence of universal virulence strategies employed by different pathogens and the conservation of innate immunity across phyla, anti-infective screening using the *C. elegans* model has the potential to identify hits that are efficacious against different groups of pathogens.

Interestingly, extracts from *N. fruticans* (UE-01-4) and *O. stamineus* (UE-12), plants widely found in South East Asia, both demonstrated good anti-infective effects. Although the mangrove palm is well-known for its traditional use by the local practitioners to treat different ailments in Asia, the pharmacological effect of *N. fruticans* remained poorly defined until 2011 when a group from Bangladesh reported that the leaf and stem of *N. fruticans* exhibited anti-hyperglycemic and antinociceptive activities in a mouse model [50]. On the other hand, *O. stamineus* leaf extract is widely used as an herbal remedy for kidney and urinary tract disorders. Previous studies revealed the anti-oxidant [51,52], anti-apoptotic [51], hepatoprotective [52] and gastroprotective [53] properties and also identified the major active fractions include eupatorin, sinensetin, 3′-hydroxy-5, 6, 7,4′-tetramethoxyflavone [54] and rosmarinic acid [55]. *N. fruticans* and *O. stamineus* extracts enabled the host to survive both antibiotic-sensitive and antibiotic-resistant *S. aureus* infection without interfering with bacterial viability, suggesting that these extracts possess compounds capable of targeting the host defense mechanism or attenuating bacterial virulence traits. The fact that the intestinal colonization of *S. aureus* was blocked after exposure to these extracts further consolidates the possibility that these compounds enhance the host immunity to eliminate the pathogen or target the bacteria factor/s that prevent them from accumulating in the intestine . Therefore, extracts UE-01-4 and UE-12 are potential anti-infective candidates that may act by targeting the host immune defenses. Further investigations are ongoing to identify the active compound/s that actively promotes lifespan extension in *S. aureus*-infected nematodes and the potential anti-infective underlying mechanism(s) of these candidates.

Not surprisingly, a small number of compounds accelerated the killing of nematodes by *S. aureus*. When the worms were exposed to these substances and fed with heat-killed *E. coli* OP50, a significant decrease in survival was observed as early as 24 hrs post-treatment compared to the untreated control implying that the substance is toxic to the host (Additional file 5). Hence, by using this *C. elegans* screen, we are able to filter compounds that exhibit toxicity upon the host [56].

## Conclusion

The results of the present study demonstrate the ability of this liquid-based screen to identify anti-infective substances that enhance host survival without interfering with bacterial viability. There is an ever-growing list of Gram-positive, Gram-negative and fungal pathogens that are known to infect *C. elegans*, many of which are of clinical relevance. With minor modifications and optimization, this platform can be adapted to screen for novel therapeutic agents against infectious agents. Furthermore, by utilising readily available mutant and reporter worm strains, the mechanism of action associated with the increased survival upon treatment can be further investigated.

## Abbreviations

NCTC: National collection of type cultures; ATCC: American type culture collection; CFU: Colony forming units; MRSA: Methicillin-resistant *Staphylococcus aureus*; DMSO: Dimethyl sulfoxide; TS: Tryticase soy; LB: Luria Bertani; NGM: Nematode growth medium; BHT: Butylated hydroxytoluene; MIC: Minimum inhibitory concentration; MBC: Minimum bactericidal concentration; TDmean: Mean time to death.

## Competing interests

The authors declare that they have no competing interests.

## Authors’ contributions

CK conceived and designed the experiments, performed the experiments, analyzed the data and prepared the manuscript including revisions. WAY and NAR were involved in synthesis of compounds and manuscript preparation. MWT and SN participated in the experimental design, data analysis and manuscript preparation. All the authors have read and approved the final manuscript.

## Pre-publication history

The pre-publication history for this paper can be accessed here:

http://www.biomedcentral.com/1472-6882/14/4/prepub

## Supplementary Material

Additional file 1 The source and voucher number of plant materials/marine sample for all extracts provided by the Institute for Pharmaceuticals and Neutraceuticals Malaysia (IPharm) and Universiti Malaysia Terengganu. Click here for file

Additional file 2 **Detailed information of all natural extracts and synthetic compounds used in the anti-infective screening.** Information of all natural extracts and synthetic compounds were presented along with the results summary of disc diffusion assay and MIC test. Click here for file

Additional file 3 **Screening protocol.** (A) Summary of the anti-infective liquid screen towards *S. aureus* in a *C. elegans* model used in this study, (B) the combination agar-liquid screen protocol used by Moy et al. [8] to screen for antimicrobials towards *E. faecalis* in a *C. elegans* model and (C) the high-throughput liquid-based chemical screen to screen for compounds that attenuate *P. aeruginosa* virulence and rescue *C. elegans* from infection [11]. Click here for file

Additional file 4 **Extract UE-01-4 had no effect on the pharyngeal pumping rate of the infected worms.** The pharyngeal pumping rates of *S. aureus*-infected nematodes upon treatment with UE-01-4 (200 μg/mL) were enumerated and compared to the untreated control. The bars correspond to mean ± SD of the pharyngeal pumps/second from one representative of two individual replicates. No significant difference in the pumping rates between the treated and untreated worms (*p* > 0.005) was observed. Click here for file

Additional file 5 **Representative compounds that were toxic towards nematodes.** Survival curves of *C. elegans* fed on heat-killed *E. coli* OP50 in the presence of two selected compounds at 200 μg/mL. Survival of OP50-fed worms reduced significantly upon exposure to UC-13 and UC-24 (*p* < 0.0001). The graph shows the mean ± SD of three replicates (10–15 animals) from a representative of two independent assays. Click here for file
