# Supplementary figures and images for: Discovery of potential anti-infectives against Staphylococcus aureus using a Caenorhabditis elegans infection model
Source: BMC Complement Altern Med. 2014 Jan 6;14:4. doi: 10.1186/1472-6882-14-4 (PMC3893568; doi:10.1186/1472-6882-14-4)

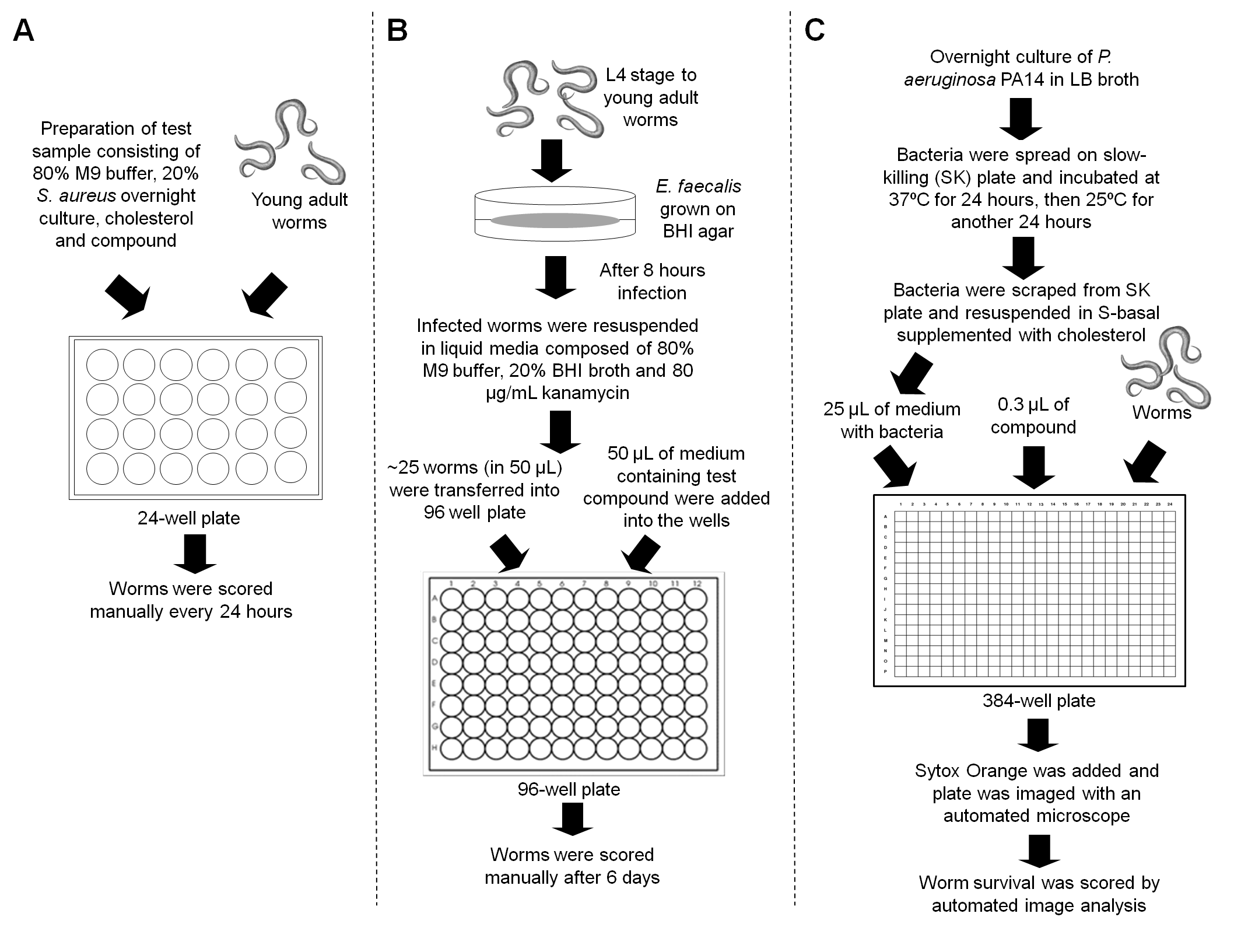

Supplement: Additional file 3 — Screening protocol. (A) Summary of the anti-infective liquid screen towards S. aureus in a C. elegans model used in this study, (B) the combination agar-liquid screen protocol used by Moy et al. [8] to screen for antimicrobials towards E. faecalis in a C. elegans model and (C) the high-throughput liquid-based chemical screen to screen for compounds that attenuate P. aeruginosa virulence and rescue C. elegans from infection [11]. [file 1472-6882-14-4-S3.tiff]

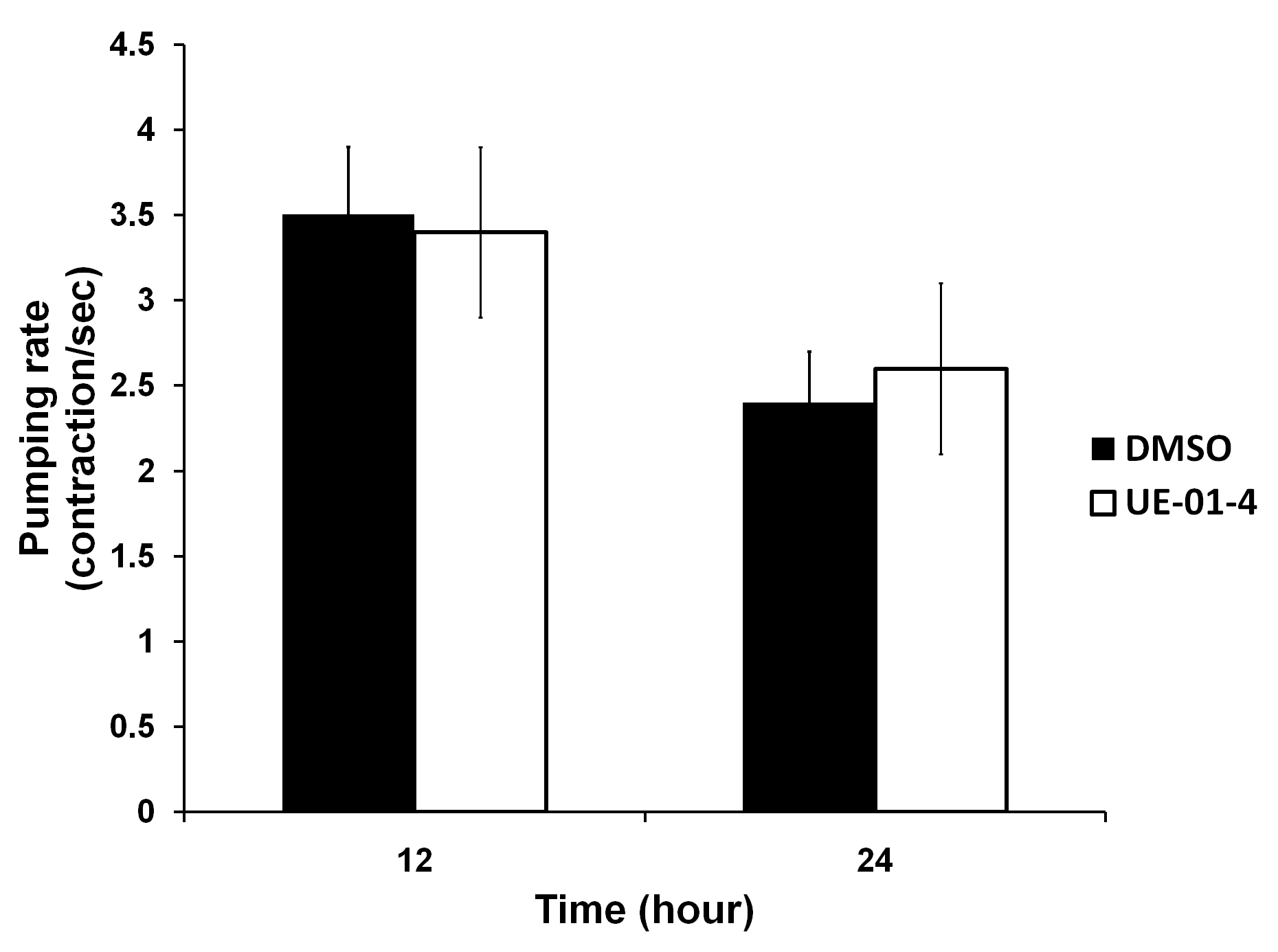

Supplement: Additional file 4 — Extract UE-01-4 had no effect on the pharyngeal pumping rate of the infected worms. The pharyngeal pumping rates of S. aureus-infected nematodes upon treatment with UE-01-4 (200 μg/mL) were enumerated and compared to the untreated control. The bars correspond to mean ± SD of the pharyngeal pumps/second from one representative of two individual replicates. No significant difference in the pumping rates between the treated and untreated worms (p > 0.005) was observed. [file 1472-6882-14-4-S4.tiff]

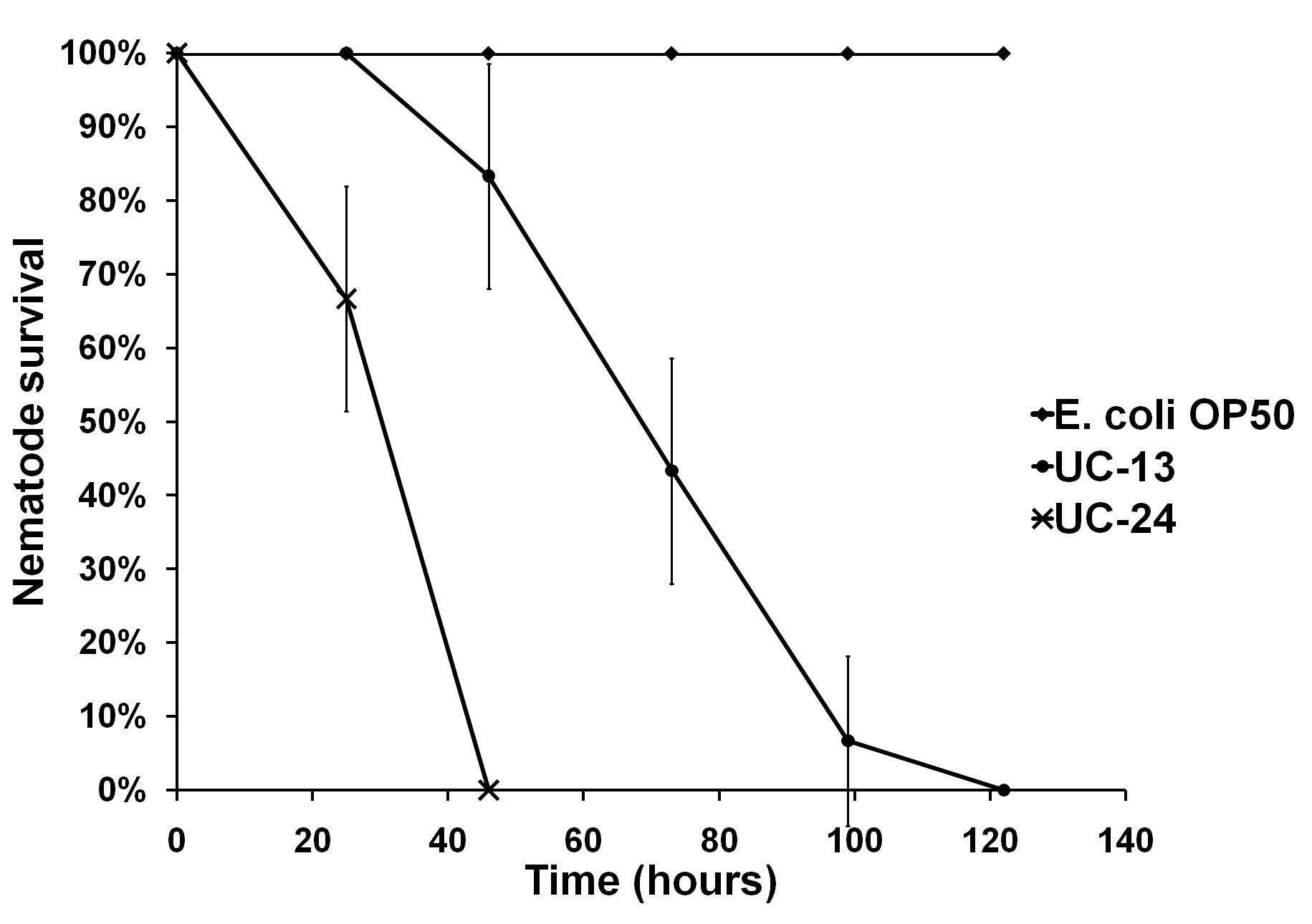

Supplement: Additional file 5 — Representative compounds that were toxic towards nematodes. Survival curves of C. elegans fed on heat-killed E. coli OP50 in the presence of two selected compounds at 200 μg/mL. Survival of OP50-fed worms reduced significantly upon exposure to UC-13 and UC-24 (p < 0.0001). The graph shows the mean ± SD of three replicates (10–15 animals) from a representative of two independent assays. [file 1472-6882-14-4-S5.tiff]
